# Direct Glass-to-Metal Welding by Femtosecond Laser Pulse Bursts: I, Conditions for Successful Welding with a Gap †

**DOI:** 10.3390/nano15151202

**Published:** 2025-08-06

**Authors:** Qingfeng Li, Gabor Matthäus, David Sohr, Stefan Nolte

**Affiliations:** 1 Institute of Applied Physics, Abbe Center of Photonics, Friedrich-Schiller-University Jena, Albert-Einstein-Str. 15, 07745 Jena, Germany; qli@thorlabs.com (Q.L.); gabor.matthaeus@zeiss.com (G.M.); stefan.nolte@uni-jena.de (S.N.); 2 Thorlabs GmbH, Münchner Weg 1, 85232 Bergkirchen, Germany; 3 ZEISS Photonics & Optics, ZEISS Microoptics, Carl Zeiss Jena GmbH, Carl-Zeiss-Promenade 10, 07745 Jena, Germany; 4 Fraunhofer Institute for Applied Optics and Precision Engineering IOF, Albert-Einstein-Str. 7, 07745 Jena, Germany

**Keywords:** femtosecond laser, micro-welding, ultrafast processing, glass–metal bonding

## Abstract

We report on the welding of optical borosilicate glass to an unpolished copper substrate (surface Ra of 0.27 µm and Rz of 1.89 µm) using bursts of femtosecond laser pulses. The present paper puts forth the hypothesis that glass–metal welding with a gap is contingent upon the ejection of molten jets of glass. We have ascertained the impact of pulse energy and focal position on weldability. This finding serves to substantiate our initial hypothesis and provides a framework for understanding the conditions under which this hypothesis is applicable. Under optimal conditions, but without the assistance of any clamping system, our welded samples maintained a breaking resistance of up to 10.9 MPa.

## 1. Introduction

Welding is an indispensable manufacturing process in almost every industrial field. In the last decade, due to the availability of ultra-short pulsed (USP) lasers with both sufficiently high pulse energy and repetition rate, a new welding process has become available [1,2]. With advantages such as high spatial selectivity, low thermal residual effects, and micron-dimension processing, it has quickly become a popular alternative technique for the welding of brittle materials (e.g., glass [3], ceramic [4]) and dissimilar materials (e.g., glass–metal [5], glass–silicon [6], glass–SiC [7], and silicon–metal [8]).

However, among all those proof-of-principle demonstrations, direct contact (the so-called optical contact) is required. To overcome this prerequisite and adapt local laser welding for mass production and industrial applications, USP laser welding with a gap at the glass-to-metal interface has been intensely investigated [9,10,11,12,13,14,15,16].

For the glass-to-glass configuration, the gap bridging mechanisms are confirmed by two independent experimental results published in 2015 [9,11], and can now be clearly summarized as necessary conditions: (1) thermal accumulation creates a sufficiently large heat-affected zone (HAZ), (2) the laser focus position is close enough to the interface so that the molten material within the HAZ can reach the interface, and (3) the ejection or mixing of the molten material fills and forms a continuous structure within the gap. The maximum bridged gap was about 16 µm, using a combination of a CW laser and an USP laser [17].

For the glass-to-metal configuration, the gap bridging mechanisms are not clear so far. Contradictory results have been reported from different groups: In 2015, Zhang et. al. reported femtosecond laser welding of alumina silicate to copper, steel and aluminum welding with a pulse duration of 160 fs and a repetition rate of 1 kHz [13]. The samples are mirror-polished, however without clamping. Under this repetition rate, thermal accumulation and the HAZ in glass is negligible, and they have attributed the adhesion to the metal particles produced during the laser ablation. They also claimed that the maximum bridgeable gap size depends on the stacking height of the ablated particles. Glass-to-metal welding under high repetition rate has also been reported [12,18]. In [18], soda-lime welding to unpolished copper without clamping was reported. In this experiment a laser with 550 fs pulse duration and 1 MHz repetition rate was used. However, even though thermal accumulation in glass is very likely under this repetition rate, they attributed the adhesion to the ablation-induced metal particles as well. In [12], with clamping, SiO2 and BK7 are welded to aluminum alloy with 5.9 ps laser at 400 kHz. With the help of a full range of electron microscopy methods [19] they discovered that the volume of the metal melt is much smaller than the volume of the glass melt. Contradictory to the first interpretation, they believe that having the molten glass fill the gap is crucial in glass-to-metal welding. This explanation is also supported by a study of the influence of focusing conditions on the welding of glass to unpolished metals across various material pairings [15]. They find that welding is enabled by the use of a low numerical aperture, which they associate with nonlinear absorption in glass, bridging a gap of up to 10 µm.

Alternative processes that enable USP glass-to-metal welding while avoiding the need for tedious sample preparation for achieving optical contact include welding metal foils [20,21] and using solder [22]. However, these methods are strongly limited with regard to the sample geometry and environments in which the use of different materials is restricted, such as in vacuum or medical applications.

In this paper, we aim to weld glass onto an unpolished copper surface without any clamping. However, the aforementioned contradictory interpretations left open which parameters to choose for optimized welding conditions. Therefore, we start our work by further examining the gap bridging mechanisms to discover the prerequisite conditions for successful glass-to-metal welding. Based on these findings, we then investigate the processing windows for achieving such conditions and we demonstrate welding of borosilicate optical glass and a surface-unpolished copper substrate (Ra of 0.27 µm and Rz of 1.89 µm).

### Conditions for Successful Welding

Before investigating the conditions for successful USP laser glass-to-metal welding, it is worth recalling some general concepts of welding. According to the International Organization of Standardization (ISO) Standard R 857 (1968) [23], welding can be stated as “an operation in which (material) continuity is obtained between parts for assembly, by various means”. R.W. Messler has pointed out in his textbook [24] that, most importantly in this definition, is the need for materials to be joined together to form one continuous body. Therefore, from the material scientist’s point of view, welding must include the formation of primary chemical bonds through the combined action of heat and pressure. Consequently, conventional welding is restricted to joining materials from the same fundamental class given that the type of bonding in each class is different, i.e., metallic bonding in metals, ionic or mixed ionic–covalent bonding in ceramics and glasses. However, thanks to a series of investigations by Pask et. al. [25,26,27], we know that continuous electronic structures across glass–metal interfaces can still be established through primary chemical bonds. In these cases, chemical bonding across the interface is achieved by having a stable thermodynamic equilibrium in the interfacial zone and having both phases saturated with an oxide of the metal substrate. At the atomic level, this condition corresponds to the presence of a metal oxide layer at the interface which is compatible and bonds with both phases. Thus metals, e.g., Mg, Zr, Mo, Ti, Fe, Cr, Ni, and Cu, which have a strong affinity for oxygen, can theoretically adhere very well to glass through mixed ionic–covalent bonding (e.g.,–Cu–Cu–O–Si–O–). In contrast, the bonding of glass to nonoxidizable metals (e.g., Au and Ag, which cannot form oxides readily) is dominated by the Lifshitz–van der Waals (LW) interaction (e.g., –Au–Au··········O–Si–O–), and generally very weak [28].

Therefore, to achieve good welding, or in other words good continuity between glass and metal, the following prerequisite conditions should be satisfied: (1) samples can achieve close contact, (2) the metal is oxidizable, and (3) high temperature and pressure reaction conditions are ensured. Given the λ/4 flatness is now an industry standard for the preparation of optical glasses, when the oxidizable metals are polished with submicron diamond suspensions and have a mirror finish with an Ra of <100 nm, by clamping an optical glass onto the polished metals, the so-called optical contact condition can be achieved before the welding. Finally, thanks to USP laser-induced localized high temperature and pressure conditions, welding with good quality can be achieved [5]. However, for unpolished metals without clamping, the approaches to achieve the aforementioned conditions, especially the first one, are not trivial. In the following section, the USP laser processing windows for achieving such conditions are investigated.

## 2. Methodology

### 2.1. Glass-to-Metal Welding Setup

For the USP laser glass-to-metal welding experiments, we use a prototype Trumpf TruMicro 2030 Femto Edition laser which has a wavelength λ of 1030 nm, a pulse duration τ of 400 fs, a repetition rate between 250 kHz and 2 MHz, a pulse energy of up to 20 µJ, and a beam quality factor $M^{2}$ of 1.12. This prototype laser can also work in burst operation mode. The temporal distance between the individual pulses within a burst is 20 ns, and the maximum number of pulses per burst is 8. Zimmermann et al. [29] show that burst mode operation can help to reduce the stress in the surroundings of the laser-induced weld seams and thus improve the welding result. This can be understood in terms of the nonlinear propagation and energy deposition in glass: Bursts of laser pulses enable a more localized energy deposition and higher resulting energy densities due to reduced plasma shielding compared to a single pulse with the same overall energy [30]. For this reason we use the burst mode with the maximum number of 8 pulses with a burst repetition rate of 125 kHz for our experiments. The pulse energies stated in this paper refer to the energy of a single pulse within the burst.

**Figure 1:**
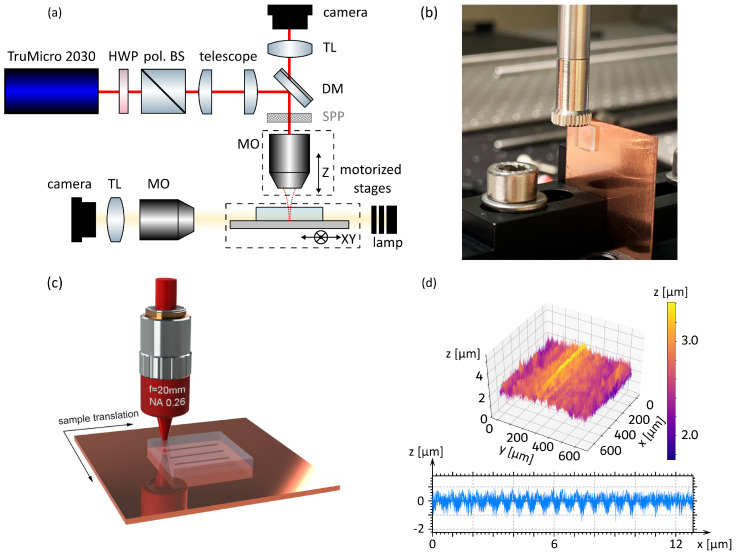
(**a**) Experimental setup for the glass-to-metal laser welding process. The pulse energy is adjusted by a half-wave plate (HWP) in combination with a polarizing beam splitter (pol. BS). A telescope is used to fully illuminate the back aperture of the microscope objective lens (MO) that is used for processing. By imaging the light that is reflected back through the same MO, a dichroic mirror (DM), and a tube lens (TL) onto a camera, we control the focus position on the sample. Additionally, through the horizontal lamp illumination, a transverse microscope is set up to monitor the formation of the welding seams. The spiral phase plate (SPP) is not used here and only added for further experiments that are presented in the second part of our mini series in this special edition [31]. (**b**) Photo of the shear force testing setup. The other end of the indenter is connected to the force gauge and the test stand. (**c**) Schematic of the welding process. The laser pulses are focused at the interface between the glass and metal by an microscope objective lens. The sample is translated with respect to the laser focus by a high-precision positioning system to realize different welding geometries. (**d**) The surface roughness measured by a confocal laser scanning microscope (CLSM).

The complete experimental setup is sketched in Figure 1a. The applied pulse energy can be adjusted by a half-wave plate (HWP) and a polarizing beam splitter (pol. BS). The output laser beam, with a diameter of 5 mm, is adapted by a telescope to fully utilize the numerical aperture of the microscope objective (MO) that is used for processing. The objective (Mitutoyo, M Plan Apo NIR, 10×, $N\;A_{\mathrm{obj}}=0.26$) is mounted on a stage allowing its displacement along the optical axis z and place the focus at the desired position for the processing. The samples are moved laterally by a xy positioning system (ALS 130, Aerotech, Pittsburgh, PA, USA). While in a further paper in this special edition [31] we examine the influence of different beam shapes on the welding results, here we only use a Gaussian beam with a waist radius $w_{0}$ of 1.86 µm for processing.

In our experiments, we can perform three-dimensional in situ monitoring of the welding process by means of two customized microscopy diagnostics. The first is a reflective microscope, which is utilized for determining the relative position of the focus with respect to the sample interface by performing surface damage scans at energies near the breakdown threshold. The second is a transversally oriented transmission microscope relying on white light illumination. The light passes through the sample along the *x* axis, and images of the welding seams in the yz plane are recorded by another objective lens (M Plan Apo, Mitutoyo, Kawasaki, Japan 10×) associated with its corresponding tube lens and camera.

To achieve comparable results with what has been reported by other groups [12,13,18], the copper samples (99.99% pure) used in this paper were sourced by Goodfellow in half-hard temper condition without further polishing. As shown in the upper image of Figure 1c, the arithmetic average value of the surface roughness (Ra) is measured as 272 nm. As the samples are unpolished, periodic serrated traces are left on the copper surface from the grinding process, as shown in the lower image of Figure 1d. The average value of the maximum peak to valley height (Rz) over the assessment length (12.9 mm) is measured as 1.89 µm. The glass samples used in this paper are 5 mm × 5 mm × 1 mm borosilicate glass (BOROFLOAT^®^ 33, Schott, Mainz, Germany; called B33) with λ/4 flatness. As presented in Figure 1b, during the welding processing the glass plate was placed upon the copper without clamping.

After the welding, we evaluate the joining strength of the welds by measuring the shear breaking strength of the welded sample. A photo of the test stand used in this paper is presented in Figure 1d, the downward thrust force was applied onto the welded glass through an indenter (cylindrical shape, 7.4 mm diameter), which was oriented parallel to the interface. The shear joining strength was estimated by the quotient of the critical thrust force at which the glass was separated from the metal and the welding area.

### 2.2. Lidt Evaluation of the Samples

**Figure 2:**
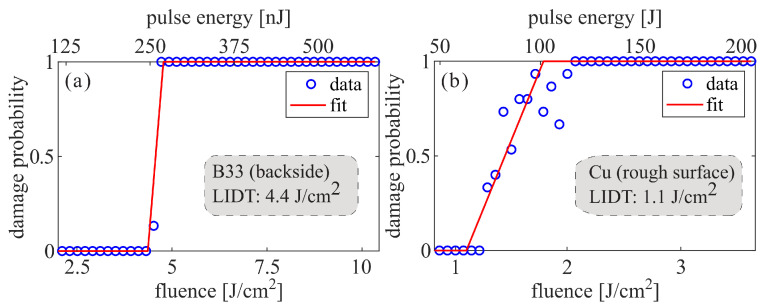
10,000-on-1 laser-induced damage threshold of (**a**) B33 glass sample, and (**b**) Cu sample.

To distinguish the processing windows in which the laser ablation of the metal or the laser-induced glass melting is predominant, and to anchor the parameters study, ahead of the welding, we performed laser-induced damage threshold (LIDT) measurements at the front surface of the copper and the back surface of the B33 glass. When there is negligible heat transfer from the metal to the glass the LIDT should represent the lowest fluence at which material can be ejected from either metal or glass. We used the same laser configuration as for the welding experiments, i.e., a burst mode with 8 pulses per burst and a burst repetition rate of 125 kHz, and each site was irradiated by 10,000 bursts. The damage probability curves for each sample are presented in Figure 2. The LIDT for the 0% damage probability given in terms of the pulse peak fluence is measured as 4.4 J/cm^2^ for B33 and as 1.1 J/cm^2^ for Cu. 

## 3. Results and Discussion

### 3.1. The Influence of the Pulse Energy

As we have mentioned in Section 1, according to the currently available literature, there are two contradictory interpretations of the USP laser glass-to-metal welding with a gap, depending on what material predominantly fills the gap.

To figure out which interpretation is closer to the nature of the successful welding, before conducting the welding experiment, we need to first find the processing regime in which the laser ablation of the metal is predominant and there is no laser-induced melting of the glass. Furthermore, to achieve comparable results, the focal position is set as 20 µm below the glass–copper interface. Under this focal condition, the LIDTs are measured at the front surface of the copper and the back surface of B33 samples. Using the LIDT values as the anchors, three different regions in terms of input pulse energy are defined: (1) below copper LIDT, (2) higher than copper LIDT but lower than B33 LIDT, and (3) higher than B33 LIDT. With the same scan speed (1 mm/s) and line interval (20 µm), the welding experiments are performed for regions (2) and (3).

**Figure 3:**
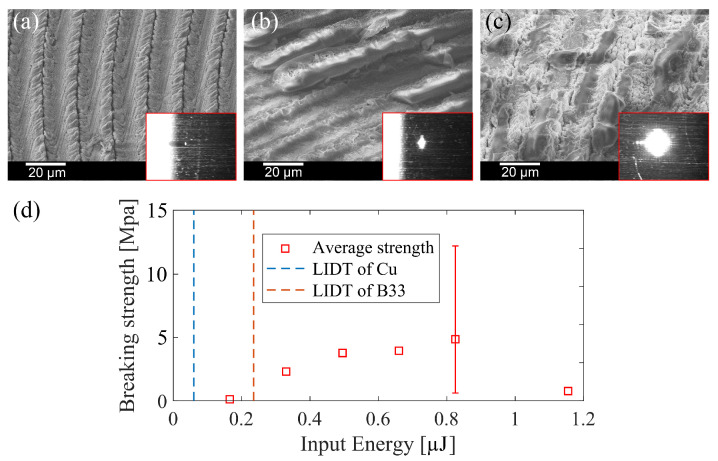
SEM images of copper surface after the separation of the welded samples under different pulse energies (**a**) 68% of the glass LIDT (0.24 µJ), (**b**) 275% of the glass LIDT and (**c**) 480% of the glass LIDT. The scan pattern used for those samples was a continuous spiral, with an inner diameter of 0.5 mm, an outer diameter of 1 mm, and a line interval of 20 µm. The insert sub-figures are the reflective microscopy snapshots recorded during the welding process. (**d**) The average breaking strength of the welded sample under different input pulse energies. The dashed lines indicate the LIDT of Cu (0.06 µJ) and B33 (0.24 µJ) when the laser is focused at 20 µm below the interface. The typical standard deviation of all data points is shown only for the one with the highest strength.

Figure 3a–c show the SEM characterizations of the welding seams after the separation under different input pulse energies. In Figure 3a the input pulse energy is high enough to create significant ablation on the Cu surface, however it is not sufficient to melt the glass. From the SEM image, one can clearly see the ablation tracks on the copper surface with ablated particles stacking beside. According to the theory proposed by Zhang et al. [13] and Matsuyoshi et al. [18], the stacking of the micro/nanometer-size metal particles/debris produced by laser ablation of the metal will attach to the glass substrate and can act as the adhesive of the welding. However, the breaking strength in this case is only 0.19 MPa in our experiments. This weak strength indicates that, with the ablated metal particles alone, welding cannot be achieved with acceptable quality. According to the breaking strength tests presented in Figure 3d, significant bonding can only be achieved when the input pulse fluence exceeds the glass LIDT. From the SEM image in Figure 3b one can clearly see that residual glass blocks stand exactly upon the scanning tracks. This resolidified glass directly proves that under this processing condition, there is molten glass wetting the copper surface and forming real contacts between the previously separated samples. The average breaking strength measured under this input pulse energy is 4 MPa. It is worth noting that, without further optimization (varying focus position, pulse energy, etc.), this value is already twice the highest breaking strength per unit area reported by previous studies in which the ablated metal particles/debris acted as adhesives [13,18]. Finally, as presented in Figure 3c, when the input pulse energy is too high (1.16 µJ, 480% of the glass LIDT), the glass seems to be affected by internal mechanical damage, so that extensive fragments of the glass remain connected to the metal, but the breaking strength decreases to 0.84 MPa. 

From the three typical processing conditions (Figure 3a–c), we have discovered that, consistent with the discussion presented in the paragraph ’Conditions for Successful Welding’ in Section 1, successful welding is established when a continuous structure forms through the glass-weld-copper interfaces. To weld glass onto copper with a rough surface, using the ablated metal particles alone, ionic or covalent bonds are unlikely to form. Even if the stacking of the ablated particles can bridge the gap and form junctions between the substrates, given the nature that the debris-to-debris and the debris-to-substrate adhesion force is dominated by LW interaction [32,33], adhesion established through the debris is inherently weak. Continuous structures are more likely to form through the emission of liquid glass. Initially, the copper surface is wetted by the liquid glass, creating a localized intimate contact area. Within this area, the formation of the copper oxide layer is facilitated by the micro plasma induced by the USP laser. In order to achieve this condition, it is necessary to have a stable ejection process to transfer molten material from one substrate through the gap to the other substrate and bridge the gap. During this transfer process, the splashing of the liquid would form debris/particles instead of a continuous block. In the glass-to-copper welding condition, due to the glass-on-top layout and the high viscosity nature of the molten glass, it is more likely to have a stable liquid transfer by viscous flow without splashing [34,35,36].

### 3.2. The Influence of the Focus Position

Another parameter that has a strong influence on the success of welding is the focus position. For glass-to-glass welding, the favorable focal position is in the range of 6–60 times the Rayleigh length below the interface [9]. In such conditions, the molten material from the lower glass substrate can be ejected and deposited at the back surface of the upper substrate as soon as the upper tip of the modification reaches the surface. In the glass-to-metal welding condition, according to our investigations in Section 3.1, to achieve successful welding, the molten material should come from the overlying glass. In this section, we investigate the favorable focus position for the molten material ejection from the glass substrate and eventually its influence on the bonding strength.

**Figure 4:**
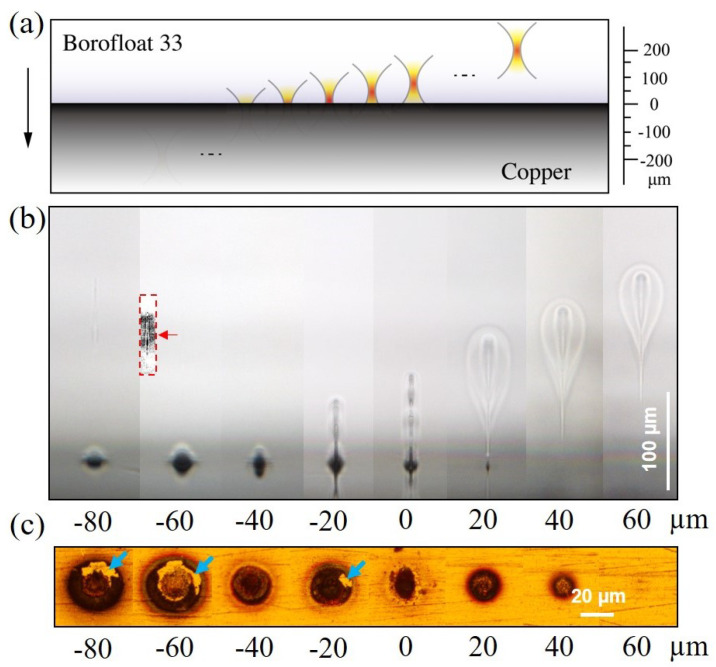
(**a**) Notation of the focal position in side view. Modifications under different focal positions in (**b**) side view in glass and in (**c**) top view on copper surface after separation. The pulse energy is 0.8 µJ and the number of bursts per site is 1000. The blue arrows indicate glass fragments stuck to the copper surface.

Firstly, as shown in Figure 4, we investigate the single site modification at different focal positions. As indicated by Figure 4a, for the notation, a focal position of 0 µm corresponds to the maximum intensity plane being located at the interface. The pulse energy is kept constant at 0.8 µJ and the number of bursts per site is 1000. Based on a homemade software [37], by taking the spherical aberration from the air-to-glass interface into account, the 1/e^2^ radius of the laser beam is calculated as 1.86 µm at the focus and the Rayleigh length is calculated as 42 µm. As shown in Figure 4b,c) when the focal position is higher than 60 µm, only the glass is modified and the molten glass is confined in the glass bulk, forming a teardrop-shaped inner structure which is corresponding to the volume of the laser-induced plasma during the laser–material interaction and an elliptical outer structure which is corresponding to the molten region. When the focal position is 20 or 40 µm above the interface, such modification structures are still preserved; however, the transmitted laser fluences are high enough in those cases to ablate the copper surfaces. When the focal position is located at the interface or 20 µm below the interface, the geometrical feature of the modifications is different. The increased voids along the inner structure and the decreased volume of the molten region indicate the emission of the molten materials. Also, for those focal conditions, we can detect the footprints of the molten glass blocks (pointed out by the blue arrows) on the copper surface, which also indicates significant wetting of the copper surface by the glass. When the focal position is lower than −60 µm, the back reflection induced modification (pointed out and highlighted in a contrast-enhanced insert in the dashed red box) starts to appear in the bulk of the glass.

**Figure 5:**
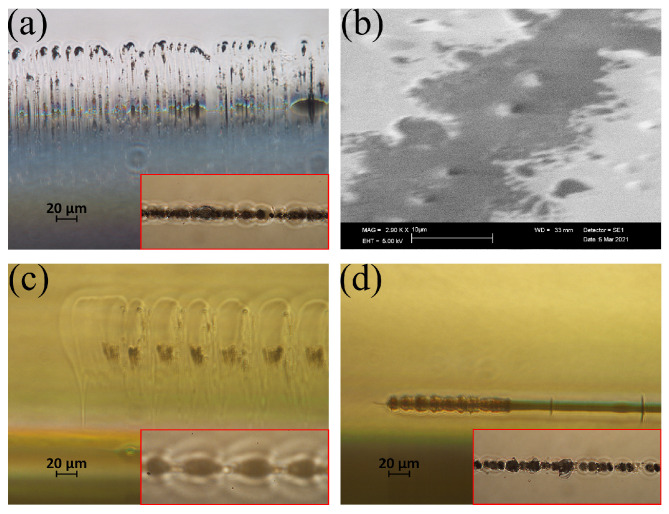
Microscopy images of the cross section of the modification in glass under different focal positions: (**a**) 0 µm, (**c**) 40 µm and (**d**) −40 µm. (**b**) SEM image of the processed glass back surface when the focal position is 0 µm. The insert images are the microscopy images of the processed glass back surfaces under the corresponding focal positions.

So far, it is known that, to transport the molten glass from the volume to the surface without unwanted absorption at the back reflection, the focal position should be in the range of 0–2 times the Rayleigh length below the glass-to-metal interface. In the actual welding scenario, the sample is moved with a translation speed of 1 mm/s. In order to confirm that, under this translation speed, there is also molten glass transportation and to get rid of the influence of back reflection, the translation processing experiments shown in Figure 5 are performed without the presence of metal. In Figure 5a,c,d, cross section images of the modification with a focal position of 0 µm, 40 µm, and −40 µm are presented. From the image insert in Figure 5c, one can directly see that when the focal position is 40 µm above the interface, the back surface of the glass remains unchanged (the shadows are the in-bulk modifications). In contrast, the insert images in Figure 5a,d clearly show that, when the focal position is at or below the interface, ablation of the back surface occurs. Figure 5b shows the SEM image of the glass back surface when the focal position is 0 µm. From the SEM image, one can see the micro-bump that is generated by the resolidified glass, and this extruded structure indicates that there is an outward transportation of the molten glass. Together with the voids presented in Figure 5a, one can confirm that, under this focal condition, there is material transfer to the free space from the backside.

**Figure 6:**
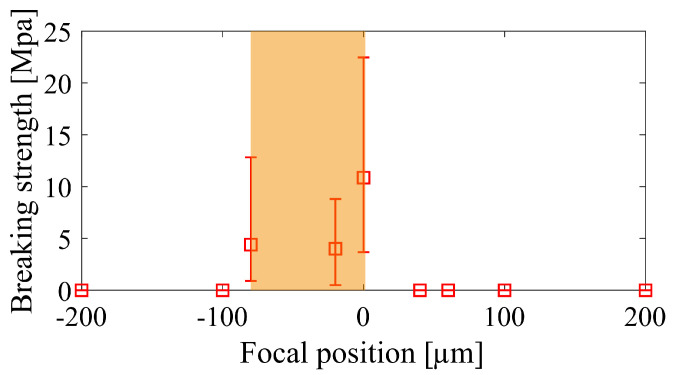
The maximum breaking strength that can be achieved for different focal positions.

Finally, we performed glass-to-copper welding under different focal conditions. The translation speed of the sample is 1 mm/s. As expected, when the focal position is 40 µm above the interface, with metal ablation alone, no significant bonding is achieved no matter how the input pulse energy adjusted. On the other hand, when the focal position is more than 100 µm below the interface it is also difficult to form significant bonding due to the back reflection. In Figure 6, we used the orange color to mark out the process window for successful welding in terms of the focal position. To make results obtained at different focal positions comparable, for each position, we first perform an energy scan to find the optimized processing energy; afterwards, with this optimized energy, we repeat the welding experiments five times and calculate the average breaking strength of the welds. The maximum value of this average breaking strength is 10.9 MPa, and it is obtained when the focal position coincides with the interface. However, it is worth noting that, due to the surface roughness and the lack of clamping assistance, the deviation of the breaking strength is large. This feature might be a problem for standardized production; however, this does not detract from its value in applications where external clamping is prohibited.

### 3.3. The Influence of the Gap Size

**Figure 7:**
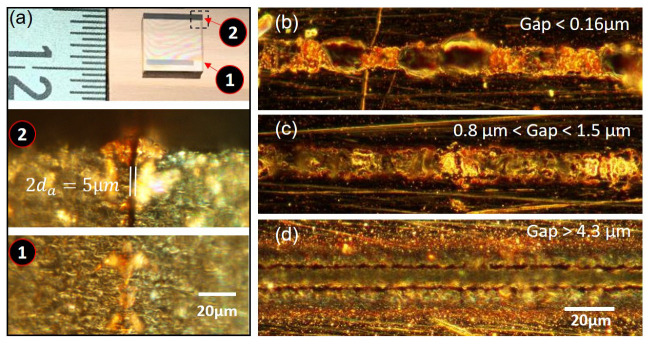
(**a**) Sample overview and light microscope images showing the gap in side view at the marked positions 1 and 2 respectively. Characteristic top view microscopy images (**b**–**d**) of the welding seam for different gap sizes: (**b**) gap < 0.16 µm, (**c**) 0.8 µm < gap < 1.5 µm and (**d**) gap = 4.3 µm.

It is worth noting that the investigations presented in the previous sections are based on the copper sample with a rough surface. However, it is actually a technical challenge to determine the micrometer gap size between glass and metal when a rough surface is present. Therefore, in this section, we chose copper samples with a mirror-polished surface (Ra 19 nm and Rz 343 nm), and determined the gap size through the air wedge method. When there is an air gap between the glass and copper, due to specular reflections of two surfaces, interference fringes, so called Newton rings, are formed. By counting the orders of the fringes, one can determine the size of the air gap. In our experiment, we further weld the narrow side of the air wedge so that we can perform ex situ characterization. As presented in Figure 7a, the direct microscopic measurement of the air wedge confirmed the gap sizes estimated by the interference method.

By setting the focal position at the back surface of the glass and performing a 500 µm long single line processing with a scan speed of 1 mm/s, we investigate how the molten glass can attach to the copper surface under different gap sizes. The pulse energy is kept at 0.8 µJ. As shown in Figure 7b,c, when the gap size is smaller than 1.5 µm, the molten glass can successfully bridge the gap and form successful bonding. After the breaking test, one can still see some attached glass knobs on the copper surface. However, when the gap is larger than 4.3 µm, the gap is so large that molten glass, as well as the ablated copper, cannot be confined to the scan tracks to form a continuous intimate contact through the melting. In the end, the maximum weldable gap size that has been tested in this paper is 1.5 µm.

Considering that the surface roughness Rz of our unpolished samples is very similar in magnitude to this limit of weldability it is not surprising that we observe such a strong variability of the resulting breaking strengths. While we could roughly determine process windows for the parameters input energy in Section 3.2 and focus position in Section 3.1, more detailed examinations of the influence of those parameters on the welding strength require polished surfaces; see part II of our welding series in this special edition [31].

## 4. Conclusions

In this study, we demonstrated the direct welding of borosilicate glass to unpolished copper using femtosecond laser pulse bursts without clamping. We found that successful welding occurs when the pulse energy exceeds the glass’s laser-induced damage threshold, allowing molten glass to wet the copper surface and form a continuous structure. The optimal focal position for welding is within 0 to 2 times the Rayleigh length below the interface, facilitating sufficient wetting of the copper surface by the glass and achieving a breaking strength of up to 10.9 MPa. Additionally, the maximum weldable gap size found for this material combination is 1.5 µm, with no significant bonding for a larger gap. Our findings support the hypothesis that the highly localized melting of the glass, along with its expansion and flow, plays a primary role in the bonding between glass and metal. In contrast, bonding based on ablated metal particles can be neglected.

## Data Availability

Data is contained within the article.
